# Supplementary material for: Risk factors for sacrococcygeal pilonidal sinus: a systematic review and meta-analysis supplemented by genetic causal assessment
Source: Front Surg. 2026 Jan 7;12:1718589. doi: 10.3389/fsurg.2025.1718589 (PMC12819706; doi:10.3389/fsurg.2025.1718589)
Supplement: Supplementary file 2 [file Datasheet2.zip › Supplementary Data 2/MR_pipeline_p5e-8/leaveone_plots/ebi-a-GCST006097.finngen_R12_L12_PILONIDALCYST.pdf]

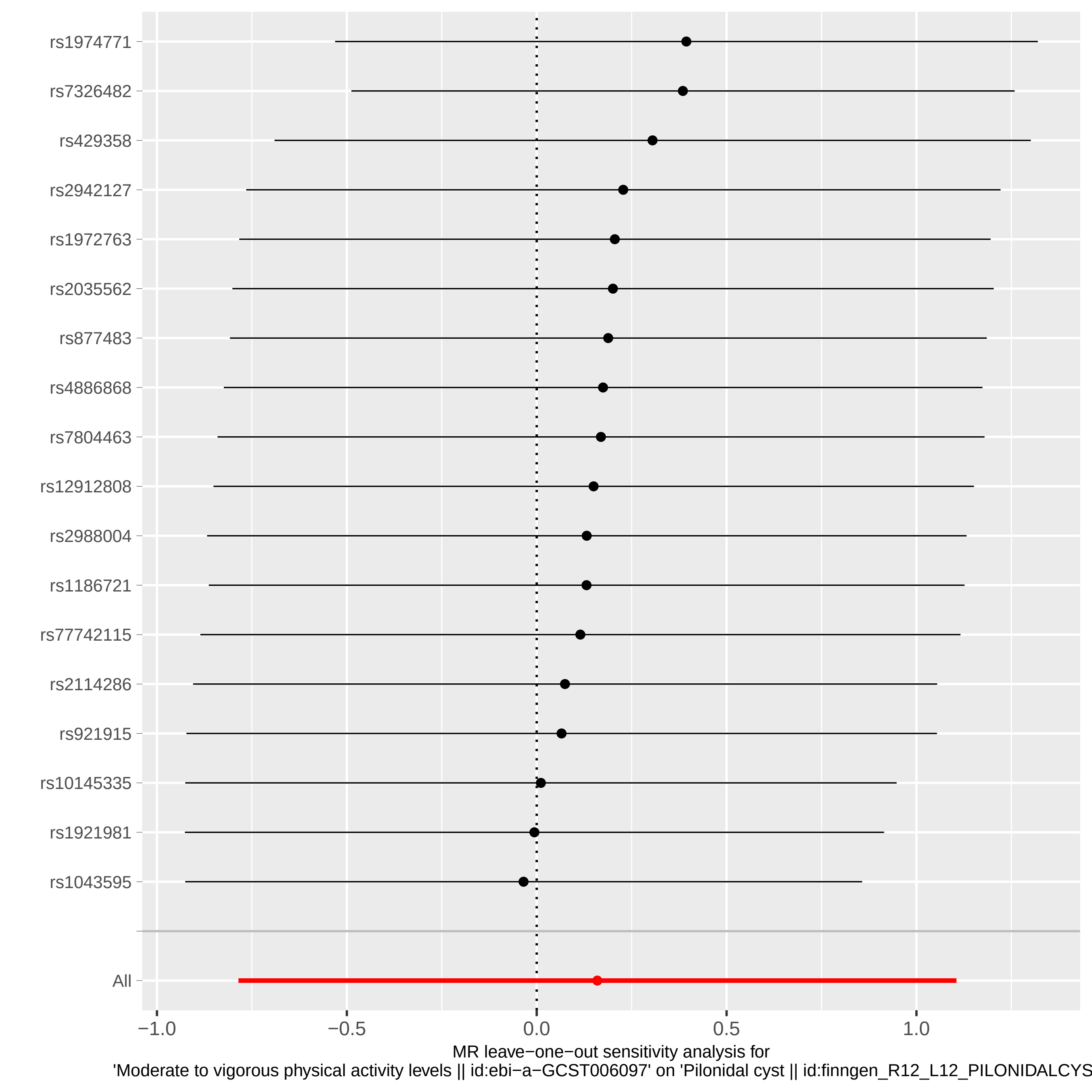
rs1974771
rs7326482
rs429358
rs2942127
rs1972763
rs2035562
rs877483
rs4886868
rs7804463
rs12912808
rs2988004
rs1186721
rs77742115
rs2114286
rs921915
rs10145335
rs1921981
rs1043595
All
−1.0
−0.5
0.0
0.5
1.0
MR leave−one−out sensitivity analysis for
'Moderate to vigorous physical activity levels || id:ebi−a−GCST006097' on 'Pilonidal cyst || id:finngen_R12_L12_PILONIDALCYS
